# Structural brain differences in school-aged children who are HIV-exposed uninfected

**DOI:** 10.1186/s12916-025-04332-3

**Published:** 2025-08-26

**Authors:** Eve A. Forster, Bilal Syed, Jennifer Bowes, Julia Young, Cassandra Kapoor, Matt Head, Jason P. Lerch, Elka Miller, Jason Brophy, Ari Bitnun, Mary Lou Smith, Lena Serghides, Margot J. Taylor, John G. Sled, the KIND Study Group

**Affiliations:** 1 Mouse Imaging Centre, Hospital for Sick Children, Toronto, Canada; 2 Translational Medicine Program, Hospital for Sick Children, Toronto, Canada; 3 Neurosciences & Mental Health Program, Hospital for Sick Children, Toronto, Canada; 4 Children’s Hospital of Eastern Ontario Research Institute, Ottawa, Canada; 5 Department of Psychology, Hospital for Sick Children, Toronto, Canada; 6 Department of Medical Imaging, Children’s Hospital of Eastern Ontario, Ottawa, Canada; 7 Department of Medical Biophysics, University of Toronto, Toronto, Canada; 8 Wellcome Centre for Integrative Neuroimaging, Department of Clinical Neurosciences, FMRIB, University of Oxford, Oxford, Nuffield UK; 9 Department of Diagnostic & Interventional Radiology, Hospital for Sick Children, Toronto, Canada; 10 Department of Medical Imaging, University of Toronto, Toronto, Canada; 11 Division of Infectious Diseases, Children’s Hospital of Eastern Ontario, Ottawa, Canada; 12 Department of Pediatrics, University of Ottawa, Ottawa, Canada; 13 Department of Pediatrics, University of Toronto, Toronto, Canada; 14 Division of Infectious Diseases, Department of Pediatrics, Hospital for Sick Children, Toronto, Canada; 15 Department of Psychology, University of Toronto Mississauga, Mississauga, Canada; 16 Toronto General Hospital Research Institute, University Health Network, Toronto, Canada; 17 Institute of Medical Sciences, University of Toronto, Toronto, Canada; 18 Women’s College Research Institute, Toronto, Canada; 19 Department of Immunology, University of Toronto, Toronto, Canada

**Keywords:** Antiretroviral therapy (ART), Brain volume, Children who are HIV-exposed uninfected (CHEU), Cognitive development, Cortical thickness, Language development, Magnetic resonance imaging (MRI), Neuroanatomical development, Sex differences

## Abstract

**Background:**

Antiretroviral therapy (ART) has dramatically reduced perinatal HIV transmission, leading to a growing population of children who are HIV-exposed but uninfected (CHEU). While the neuroanatomic developmental impacts of in utero HIV and ART exposure have been studied in young children, long-term effects on school-aged children are poorly understood, prompting this investigation.

**Methods:**

Fifty-eight CHEU and 38 children who are HIV-unexposed, uninfected (CHUU), 6–12 years old, were recruited through hospitals and community groups in Ontario, Canada. From T1-weighted magnetic resonance images, volume, cortical thickness, and gray-/white-matter tissue volume were extracted. Multiple linear regression models controlling for sex, age, household income, and total brain volume were fit to assess differences by in utero HIV exposure, with additional sex-stratified analyses to uncover sex-specific effects.

**Results:**

Compared with CHUU, CHEU showed total brain volumes that were significantly smaller by 49.7cm^3^ (95% CI [− 95.66, − 3.67]) and cortices thinner by 0.08 mm (95% CI [− 0.13, − 0.02]). In male CHEU, three regions displayed volumetric age-exposure interactions: the bilateral pars opercularis at 0.36 cm^3^/year (95% CI [0.10, 0.62]), left rolandic operculum at 0.22 cm^3^/year (95% CI [0.04, 0.39]) and left precentral gyrus at 0.71 cm^3^/year (95% CI [0.22, 1.21]), suggesting delayed maturation in those regions. Bilateral frontal lobe cortical thickness was reduced by 0.07 mm in CHEU (95% CI [− 0.14, − 0.006]), most pronounced in the left orbital middle frontal gyrus with a reduction of 0.20 mm among male CHEU (95% CI [− 0.32, − 0.07]). An age-exposure interaction of 0.06 cm^3^/year in bilateral amygdala volume (95% CI [− 0.11, − 0.01]) suggested reduced growth or altered developmental trajectory among CHEU, whereas male CHEU showed bilateral hippocampal volumes diminished by 0.21 cm^3^ (95% CI [− 0.40, − 0.01]).

**Conclusions:**

These findings suggest that in utero HIV and ART exposure have broad neuroanatomic developmental impacts, particularly in boys, with significant differences in brain regions critical for motor function, expressive language, memory, and emotion. These structural differences align with previously reported motor and language deficits and highlight the importance of early intervention and tailored support strategies for CHEU.

**Supplementary Information:**

The online version contains supplementary material available at 10.1186/s12916-025-04332-3.

## Background

The successful implementation of public health programs promoting universal access to antiretroviral therapy (ART) has significantly reduced perinatal human immunodeficiency virus (HIV) transmission, leading to an increase in the number of children who have been exposed to HIV in utero but not infected. In the USA, approximately 100,000 children were exposed to ART in utero between 1994 and 2010, preventing an estimated 22,000 cases of perinatal HIV transmission [1]. In Canada, almost 6000 infants have been exposed to HIV in utero since 1990, and in 2022, the perinatal transmission rate for those who received 4 or more weeks of combined ART during pregnancy was 0.6%, compared to 23.5% who did not [2]. Globally, the number of children who are HIV-exposed and uninfected (CHEU) has reached 16 million [3].

Previous research has established that in utero exposure to HIV and ART affects early neurodevelopment. Decreases in expressive language and motor (but not cognitive) development were reported in 1-year-old CHEU [4]. Cognitive and motor (but not language) delays were reported in CHEU at 12–14 months [5], as well as lower language (especially expressive language) and gross motor development scores in 2-year-olds [6–8]. At 3 years, and more so at 5 years, an elevated risk of language impairments was observed in CHEU compared to population norms [9]. A separate group of 3–4-year-old CHEU scored significantly lower than children who were HIV-unexposed, uninfected (CHUU) on measures of intelligence and visuomotor function (but not language), and upon retesting 2 years later, they scored significantly lower on language as well [10]. At 5–6 years, CHEU scored lower than CHUU on general intelligence, reading, math, and visuomotor function [11, 12]. A meta-analysis of cognitive and motor development in 0–8-year-olds found CHEU lower in both [13]. In 5–12-year-olds, lower cognitive, memory, and attention scores were observed in CHEU [14].

The underlying mechanisms and long-term impacts of these neurodevelopmental effects are less clear, motivating further study. In animal models of in utero ART exposure in the absence of HIV infection, decreases or deficits in attention, memory, grooming, and social interaction have been reported, suggesting that both HIV and ART contribute to the development of these deficits [15, 16]. Diffusion tensor imaging studies at 2–4 weeks and 5 and 7 years have found white matter abnormalities in CHEU [17, 18], but evidence is mixed, and some have found no association [15]. At 2–6 weeks of age, CHEU had lower gray matter volume (especially if their birth parent had a low CD4 count) and lower caudate volume [19]. Wedderburn et al. [20], a South African cohort study of 2–3-year-olds, has reported that CHEU display thicker prefrontal cortices, especially in the medial orbitofrontal region, but research on older age groups is lacking. Our study investigated the replicability of these findings in an older Canadian cohort, using a similar approach to assess whether these effects persist in older children.

Addressing this knowledge gap could guide early educational interventions and inform healthcare policy to better support the developmental needs of CHEU. In support of this goal, we investigated the effects of in utero HIV and ART exposure on brain development in 6- to 12-year-old children. The primary aim was to compare cortical volume and thickness between CHEU and CHUU, to determine whether specific brain regions or structures are particularly susceptible to HIV and ART exposure.

## Methods

We used a cross-sectional design to compare the brain structure of CHEU and CHUU. The Kids Imaging and Neurocognitive Development (KIND) study was approved by the Hospital for Sick Children (SickKids), the Children’s Hospital of Eastern Ontario (CHEO), and the University Health Network Research Ethics Boards, and followed the Strengthening the Reporting of Observational Studies in Epidemiology (STROBE) reporting guidelines for cross-sectional studies (see Additional file 1: Appendix S1). It is an ongoing study of 6–12-year-olds who are either CHEU or CHUU, defined as follows. CHEU had documented in utero HIV exposure via a birth parent with documented positive HIV serostatus during the pregnancy or earlier, had at least 4 weeks of documented in utero exposure to ART, and tested negative for HIV shortly after birth and during standard follow-up. CHUU had no exposure to either HIV or ART, either pre- or postnatally. These classifications were confirmed through clinical records and caregiver reports. Children living with HIV were excluded from the study to focus on the effects of in utero HIV exposure only. Additional exclusion criteria were the presence of metal implants, contraindicated for magnetic resonance imaging (MRI); previous developmental or neurological conditions unrelated to HIV/ART exposure with residual dysfunction; and exposure to significant substance use during pregnancy such as smoking (more than 5 per day or continuing after pregnancy was recognized), alcohol (more than 1 drink per week or continuing after pregnancy was recognized), or any consumption of illicit drugs known to influence fetal neurodevelopment. Some participants experienced in utero exposure to smoking and alcohol below the exclusion criteria (reported in Table 1).

**Table 1:** Demographic and clinical attributes by exposure group

	Total (*N* = 96)	CHEU (*N* = 58)	CHUU (*N* = 38)	*p*-value
*Demographic variables*				
Child’s age at scan, years	9.0 (1.6)	9.0 (1.5)	9.0 (1.6)	0.68
Sex				0.26
Female	46 (48%)	31 (53%)	15 (39%)	
Male	50 (52%)	27 (47%)	23 (61%)	
Annual household income (Canadian dollars) ^†^				0.10
< $25,000	19 (20%)	15 (26%)	4 (11%)	
$25,000–$49,999	30 (31%)	20 (34%)	10 (26%)	
$50,000–$74,999	19 (20%)	8 (14%)	11 (29%)	
$75,000–$99,999	6 (6%)	4 (7%)	2 (5%)	
$100,000 +	20 (21%)	9 (16%)	11 (29%)	
Missing	2 (2%)	2 (3%)	-	
Main caregiver’s highest level of education ^†^				0.31
Did not finish high school	7 (7%)	5 (9%)	2 (5%)	
High school degree	19 (20%)	15 (26%)	4 (11%)	
College degree	44 (46%)	24 (41%)	20 (53%)	
University undergraduate degree	12 (12%)	7 (12%)	5 (13%)	
Post-university undergraduate degree	13 (14%)	6 (10%)	7 (18%)	
Missing	1 (1%)	1 (2%)	-	
Smoking during pregnancy (any)	8 (8%)	5 (9%)	3 (8%)	1.00
Alcohol use during pregnancy (any)	15 (16%)	8 (14%)	7 (18%)	0.75
CD4 count in pregnancy, closest to delivery (cells/mm^3^)				
< 200		2 (3%)		
200–349		2 (3%)		
350–499		5 (9%)		
500 +		11 (19%)		
Missing		38 (66%)		
Viral load in pregnancy, closest to delivery (copies/mL)				
Undetectable (< 50)		43 (74%)		
Detectable (50–999)		1 (2%)		
Virally unsuppressed (1000 +)		0 (0%)		
Missing		14 (24%)		
Timing of antiretroviral drug initiation				
Before conception		40 (69%)		
During pregnancy		14 (24%)		
Missing		4 (7%)		
Class of antiretroviral regimen, earliest recorded during pregnancy				
Protease inhibitor (PI)		26 (45%)		
Non-nucleoside reverse transcriptase inhibitor (NNRTI)		17 (29%)		
Integrase strand transfer inhibitor (INSTI)		5 (9%)		
Nucleoside reverse transcriptase inhibitor (NRTI)		2 (3%)		
INSTI/PI		2 (3%)		
INSTI/NNRTI/PI		1 (2%)		
Missing		5 (9%)		
*Perinatal measurements*				
Birth weight, kg	3.1 (0.8) *N* = 92	2.9 (0.8) *N* = 57	3.4 (0.5) *N* = 35	0.001 *
Gestational age at birth, weeks	38.7 (2.7) *N* = 94	38.0 (3.1) *N* = 57	39.3 (1.5) *N* = 37	< 0.001 *
Prematurity				0.024 *
Gestational age < 37 weeks	20 (21%)	17 (29%)	3 (8%)	
Gestational age ≥ 37 weeks	74 (77%)	40 (69%)	34 (89%)	
Missing	2 (2%)	1 (2%)	1 (3%)	
Small for gestational age				0.37
< 10th birth weight centile	10 (10%)	8 (14%)	2 (5%)	
≥ 10th birth weight centile	82 (85%)	49 (84%)	33 (87%)	
Missing	4 (4%)	1 (2%)	3 (8%)	
*Neuroanatomical measurements at scan*				
Total brain volume, cm^3^	1370.7 (131.3)	1350.0 (122.4)	1409.4 (136.1)	0.016 *

Sample size *N* (%) or median (standard deviation) are reported. Ns for summary statistics are found in the table heading unless otherwise specified. *p*-values were calculated by comparing continuous variables with unpaired *t*-tests and categorical variables with chi-square tests. Two variables^†^ were converted to integers and treated as continuous. * *p* < 0.05. *CHEU*, children who are HIV-exposed uninfected; *CHUU*, children who are HIV-unexposed uninfected

Parents of CHEU were contacted by a clinical team member at SickKids in Toronto, Ontario, or CHEO in Ottawa, Ontario, or through previous developmental studies where consent had been provided for follow-up communications about future studies. CHUU with similar socio-economic backgrounds were recruited by word-of-mouth through parents of CHEU. Some CHEU and CHUU were recruited from the “Angiogenesis and Adverse Pregnancy Outcomes in Women with HIV” cohort of pregnant women with HIV and matched pregnant women without HIV [21]. Other participants were recruited through community groups, and before- and after-school programs in areas of similar socio-economic status to the CHEU population. Data collection began in 2020 and is ongoing. Written informed consent was obtained from the primary caregiver. Participant assent was also obtained.

### Demographic variables and perinatal measurements

Demographic variables such as household income, caregiver education, and smoking and alcohol use during pregnancy were collected through a parent questionnaire. Medical records provided data on class and timing of antiretroviral regimen, CD4 count and viral load of the birth parent during the relevant pregnancy. Medical records (if available) and the parent questionnaire provided information on sex, date of birth, birth weight, and gestational age at birth. All data were cross-referenced to ensure accuracy.

Income was grouped into $25,000 increments and caregiver education into levels spaced approximately 4–5 years apart. These two variables were subsequently converted to integers and treated as continuous, as the differences between each level were relatively consistent. CD4 counts were grouped according to the World Health Organization immunological classification for established HIV infection [22]. Viral load was grouped according to World Health Organization definitions of viral suppression and low-level viremia [23].

### Neuroimaging data acquisition and processing

T1-weighted 3D magnetic resonance (MR) images were obtained on a 3-Tesla Siemens Prisma MRI at the SickKids site and a 3-Tesla Siemens Skyra MRI at the CHEO site using a 3D MPRAGE protocol (TR/TE: 1870.0/3.10 ms, FA: 9°, FOV: 192 × 240 × 256 mm, 0.8 mm isotropic voxels; scan time: 5 min).

The images were processed with CIVET software (version 2.1.1) [24] producing measures of volume, cortical thickness, and surface area of individual cortical regions. After stereotaxic registration to the Montreal Neurologic Institute ICBM152 non-linear symmetric template [25–29] and nonuniformity correction [30], the tissue was classified into white matter, gray matter, and cerebrospinal fluid. The white matter-gray matter boundary was first extracted and then expanded outwards to extract the pial surface, allowing the calculation of cortical thickness (via average t-link distance) [31, 32], volume, and surface area. Region boundaries were defined by the automated anatomical labeling atlas (AAL) labeling package [26, 33].

Parcellation of subcortical volumes was accomplished by label voting with multiple automatically generated templates (MAGeT) [34]. A template library of 21 T1 images was individually transformed by non-linear image registration to a merged subcortical atlas provided by the CoBrALab [35–37] to obtain an anatomical segmentation for that template. See Additional file 2: Supplementary Table S1 for all CIVET and MAGeT measures calculated for each participant.

The ICBM152 template is based on adult anatomy, but the key metrics derived from MAGeT and CIVET are computed in native space and do not hinge on choice of template, provided registration is successful. All registered images were visually inspected to confirm adequate registration. Separate quality control checks were performed for the CIVET and MAGeT analyses, resulting in the removal of 10 participants’ CIVET data and 1 participant’s MAGeT data. Each image was also visually inspected. After CIVET quality control checks, data from only 5 CHEO participants remained, comprising only 5% of the total sample. Due to the substantial difference in sample size at the two sites, we determined that the statistical assumptions of our models would not be met unless we limited the analyses to the SickKids site only.

### Statistical analyses

**Fig. 1:**
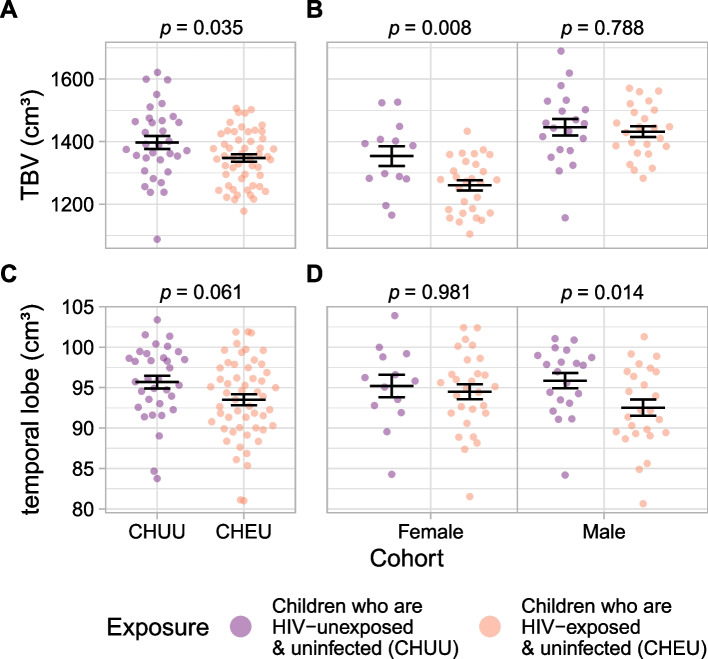
Adjusted whole brain and temporal lobe volumes, stratified by sex and exposure group. **A**, **B** Total brain volume by **A** exposure group and **B** sex and exposure group. **C**, **D** Temporal lobe volume, similarly stratified. Volumes were adjusted for sex (panels **A** and **C** only), age, income, and total brain volume (panels **C** and **D** only). Horizontal lines indicate means and standard errors. Above each plot are *p*-values of the exposure group effect

**Fig. 2:**
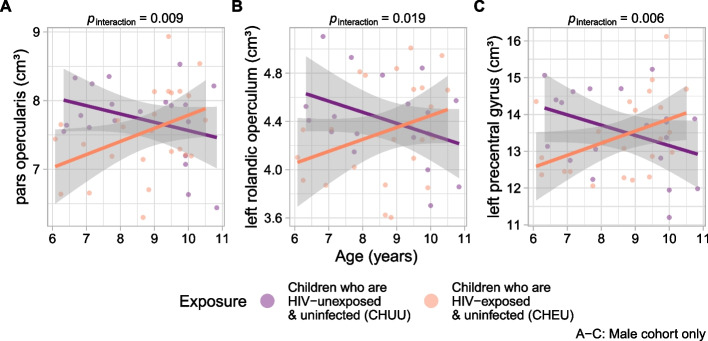
Adjusted volumes of 3 cortical regions by age and exposure group in males. Plots of volume by age, stratified by exposure group, for **A** pars opercularis, **B** left rolandic operculum, and **C** left precentral gyrus. Volumes were adjusted for income and total brain volume. Linear trends and 95% confidence intervals are also plotted. Above each plot are *p*-values for the age by exposure interaction term

**Fig. 3:**
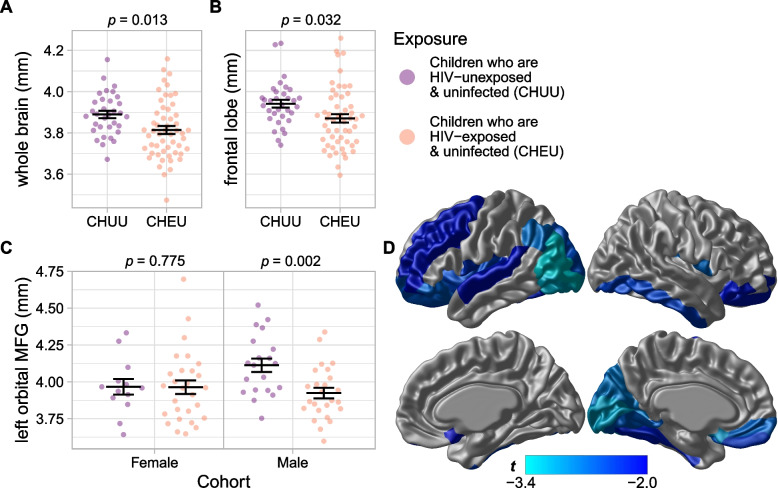
Adjusted cortical thickness, stratified by sex and exposure group. Cortical thickness of the **A** whole brain by exposure group, **B** frontal lobe by exposure group, and **C** left orbital middle frontal gyrus (MFG) by sex and exposure group. Thicknesses were adjusted for sex (panels **A** and **B** only), age, income, and total brain volume (panels **B** and **C** only). Horizontal lines indicate means and standard errors. Above each plot are *p*-values of the exposure group effect. **D** *t*-values of the exposure effect within a cortical thickness model, 15% FDR

**Fig. 4:**
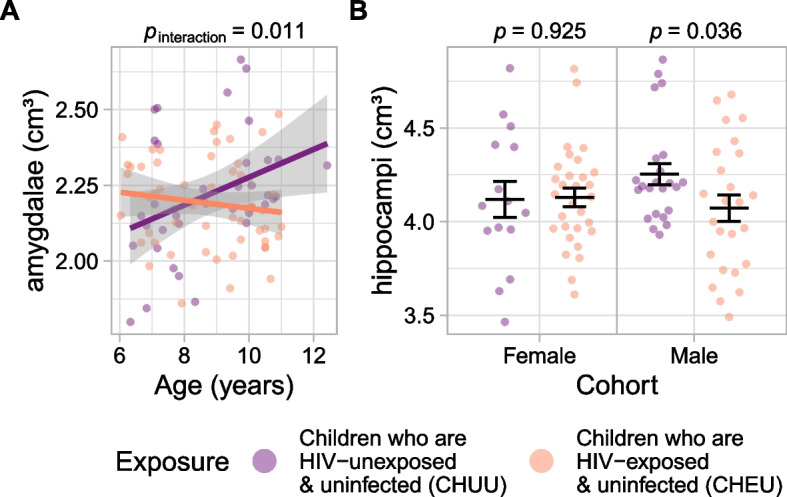
Adjusted volumes of the amygdalae and hippocampi, stratified by exposure group. **A** Volume of the amygdalae (adjusted for sex, income, and total brain volume) by age, with linear trends and 95% confidence intervals. *p*: *p*-values of the age by exposure interaction. **B** Volume of the hippocampi (adjusted for age, income, and total brain volume) by sex. Horizontal lines indicate means and standard errors. *p*: *p*-values of the exposure effect

Multiple linear regression models were constructed for the volume of each lobe and brain region, with exposure group, sex, age (centered), annual household income, and total brain volume included as covariates. Missing data were omitted. Birth weight, gestational age, and prematurity were considered to be potential mediators of the effect of in utero HIV/ART exposure on neurodevelopment, rather than confounders, and thus were not included as covariates. Two variants of this model were examined, with and without an age by exposure group interaction term. Similar models were constructed for sex-stratified analyses, omitting the sex covariate. To further examine age by exposure group interactions, the exposure group covariate was omitted from the model, allowing for separate stratified analyses within the exposed (CHEU) and unexposed (CHUU) groups to reveal age effects. Linear models of cortical thickness globally and specific cortical lobes and regions were similarly constructed. A 15% false discovery rate (FDR) was used where multiple comparisons were conducted. A specific analysis was also performed, adding caregiver education and removing total brain volume from the covariates to replicate modeling by Wedderburn et al. [20] as best as possible (all variables, except maternal age, were available within our dataset). This analysis employed the same multiple linear regression models and FDR correction as all other analyses in this manuscript, changing only the covariates as described. To visualize the impact of exposure group (without the influence of other model covariates) (see Figs. 1, 2, 3, and 4), an adjusted measure was computed for each region, subtracting the centered effects of sex, age, income, and total brain volume.

## Results

Participants included 58 CHEU (31 female, 27 male) aged 6.0–11.0 years (*mean age* = 8.7, *standard deviation* = 1.5) and 38 CHUU (15 female, 23 male) aged 6.3–12.4 years (*mean age* = 8.8, *standard deviation* = 1.6). Table 1 provides further demographic and clinical information. The two groups differed significantly for several perinatal measurements (birth weight, gestational age at birth, and prematurity).

### Total brain volume

CHEU had significantly smaller brain volumes than CHUU after controlling for sex, age, and income (estimated difference = − 49.7cm^3^, 95% confidence interval (CI) [− 95.66, − 3.67], *p* = 0.035; standardized regression coefficient (*β*) = − 0.38) (Fig. 1). The effect was present within the female cohort (estimated difference = − 95.2 cm^3^, 95% CI [− 163.86, − 26.61], *p* = 0.008; *β* = − 0.91) but not the male cohort (estimated difference = − 8.8 cm^3^, 95% CI [− 74.13, 56.61], *p* = 0.788; *β* = − 0.08) (Fig. 1).

Total brain volume was a covariate in all subsequent models, except for the replication of Wedderburn et al.’s findings [20].

### Temporal lobe

Bilateral temporal lobe volumes were smaller in male CHEU than male CHUU (estimated difference = − 3.7 cm^3^, 95% CI [− 6.61, − 0.78], *p* = 0.014; *β* = − 0.40); no effect was observed in the female cohort (estimated difference = − 0.05cm^3^, 95% CI [− 3.89, 3.79], *p* = 0.981; *β* = 0.0) (Fig. 1). Other lobes were examined but did not meet the significance threshold after FDR correction.

### Pars opercularis

An age by exposure interaction was observed in the bilateral pars opercularis (the opercular part of the inferior frontal gyrus) in the male cohort (estimated difference = 0.36 cm^3^/year, 95% CI [0.10, 0.62], *p* = 0.009; *β* = 0.66) (Fig. 2). Volumes converged over time, decreasing (non-significantly) with age in male CHUU (estimated difference = − 0.13 cm^3^, 95% CI [− 0.32, 0.05], *p* = 0.149, *β* = − 0.23), and increasing (non-significantly) in male CHEU (estimated difference = 0.21 cm^3^, 95% CI [− 0.001, 0.42], *p* = 0.051, *β* = 0.42). No interaction was found in the female cohort (estimated difference = − 0.07 cm^3^/year, 95% CI [− 0.31, 0.18], *p* = 0.580; *β* = − 0.14).

### Rolandic operculum and precentral gyrus

Two adjacent regions displayed a similar relation as the pars opercularis. An age by exposure interaction was observed in the left rolandic operculum, in the male cohort (estimated difference = 0.22 cm^3^/year, 95% CI [0.04, 0.39], *p* = 0.019; *β* = 0.61) (Fig. 2), with volumes converging over time (CHUU: estimated difference = − 0.15 cm^3^, 95% CI [− 0.30, 0.002], *p* = 0.052, *β* = − 0.38; CHEU: estimated difference = 0.11 cm^3^, 95% CI [− 0.03, 0.26], *p* = 0.116, *β* = 0.33). Another age by exposure interaction was found in the male cohort in the left precentral gyrus (estimated difference = 0.71 cm^3^/year, 95% CI [0.22, 1.21], *p* = 0.006; *β* = 0.71) (Fig. 2); volumes decreased with age in male CHUU (estimated difference = − 0.38 cm^3^, 95% CI [− 0.73, − 0.03], *p* = 0.035, *β* = − 0.37) and slightly (non-significantly) increased in male CHEU (estimated difference = 0.30 cm^3^, 95% CI [− 0.09, 0.69], *p* = 0.126, *β* = 0.30).

### Cortical thickness

CHEU had thinner cortices overall than CHUU (estimated difference = − 0.08 mm, 95% CI [− 0.13, − 0.02], *p* = 0.013, *β* = − 0.51) (Fig. 3). A small but significant difference in bilateral frontal lobe cortical thickness was found between exposure groups, with CHEU having thinner frontal cortices than CHUU (estimated difference = − 0.07 mm, 95% CI [− 0.14, − 0.006], *p* = 0.032; *β* = − 0.45) (Fig. 3). Other lobes were examined but did not meet the significance threshold after FDR correction. Thinner cortices were detected in male CHEU in the left orbital middle frontal gyrus (estimated difference = − 0.20 mm, 95% CI [− 0.32, − 0.07], *p* = 0.002, *β* = − 0.86) (Fig. 3) and the left calcarine fissure and surrounding cortex (estimated difference = − 0.19 mm, 95% CI [− 0.32, − 0.07], *p* = 0.004, *β* = − 0.83).

A replication of the findings of Wedderburn et al. [20] was also performed, modeling cortical thickness as a function of sex, age, caregiver education, income, and exposure group. This was the only model in which total brain volume was not included as a covariate, and a much larger number of regions were found to be statistically significant using this model (see Table 2). (See Additional file 3: Supplementary Table S2 for the full table of volume estimates, and Additional file 3: Supplementary Table S3 for a corresponding table of cortical thickness estimates.) Effects were found in similar areas (although in the opposite direction) as reported by Wedderburn et al. The bilateral frontal cortices of CHEU were thinner than those of CHUU (estimated difference = − 0.08 mm, 95% CI [− 0.14, − 0.02], *p* = 0.013, *β* = − 0.51). The medial orbitofrontal cortex reported by Wedderburn et al. overlaps with two regions of the AAL parcellation used in the current study: the olfactory cortex and gyrus rectus. The left and right olfactory cortices were thinner in CHEU than CHUU (left: estimated difference = − 0.20 mm, 95% CI [− 0.33, − 0.07], *p* = 0.003, *β* = − 0.63; right: estimated difference = − 0.10 mm, 95% CI [− 0.19, 0.00], *p* = 0.046, *β* = − 0.43), as was the left gyrus rectus (estimated difference = − 0.16 mm, 95% CI [− 0.30, − 0.02], *p* = 0.023, *β* = − 0.49) (Fig. 3D). The difference in the right gyrus rectus did not reach significance (estimated difference = − 0.07 mm, 95% CI [− 0.19, 0.05], *p* = 0.242, *β* = − 0.26). Broad swaths of cortex were thinner in CHEU, with the largest effects in several orbital subregions of the left superior and inferior frontal gyri, left olfactory cortex, insula, angular gyrus, and many regions across the left occipital lobe; in the right hemisphere, cortex was thinner in the rolandic operculum, inferior temporal gyrus, and inferior occipital gyrus (see Table 2).

**Table 2:** Estimated differences in cortical thickness by exposure group

Region	Model 1 (entire cohort)			Model 2 (male cohort)		
	Estimated difference (mm) [95% CI]	*p*-value	*β*	Estimated difference (mm) [95% CI]	*p*-value	*β*
*Left hemisphere*						
Rolandic operculum	− 0.07 [− 0.14, − 0.01]	0.022 *	− 0.49	− 0.09 [− 0.18, 0.00]	0.040	− 0.57
Superior frontal gyrus, dorsolateral	− 0.09 [− 0.18, − 0.01]	0.038 *	− 0.44	− 0.09 [− 0.22, 0.03]	0.125	− 0.44
Middle frontal gyrus	− 0.07 [− 0.14, 0.00]	0.039 *	− 0.42	− 0.04 [− 0.14, 0.06]	0.400	− 0.23
Superior frontal gyrus, orbital part	− 0.15 [− 0.26, − 0.03]	0.011 *	− 0.55	− 0.16 [− 0.32, 0.01]	0.060	− 0.58
Superior frontal gyrus, medial orbital	− 0.13 [− 0.22, − 0.03]	0.009 *	− 0.54	− 0.11 [− 0.25, 0.02]	0.095	− 0.46
Middle frontal gyrus, orbital part	− 0.10 [− 0.20, 0.00]	0.044 *	− 0.43	− 0.20 [− 0.32, − 0.07]	0.002 *	− 0.86
Inferior frontal gyrus, orbital part	− 0.09 [− 0.15, − 0.02]	0.010 *	− 0.51	− 0.11 [− 0.20, − 0.01]	0.031	− 0.60
Gyrus rectus	− 0.16 [− 0.30, − 0.02]	0.023 *	− 0.49	− 0.13 [− 0.34, 0.08]	0.231	− 0.36
Olfactory cortex	− 0.20 [− 0.33, − 0.07]	0.003 *	− 0.63	− 0.14 [− 0.33, 0.06]	0.170	− 0.41
Superior temporal gyrus	− 0.08 [− 0.16, 0.00]	0.041 *	− 0.44	− 0.09 [− 0.21, 0.03]	0.150	− 0.42
Heschl’s gyrus	− 0.08 [− 0.15, − 0.01]	0.032 *	− 0.46	− 0.05 [− 0.16, 0.05]	0.322	− 0.29
Angular gyrus	− 0.08 [− 0.14, − 0.02]	0.007 *	− 0.55	− 0.12 [− 0.21, − 0.04]	0.006	− 0.78
Superior occipital gyrus	− 0.12 [− 0.22, − 0.03]	0.013 *	− 0.48	− 0.09 [− 0.23, 0.06]	0.235	− 0.35
Middle occipital gyrus	− 0.13 [− 0.21, − 0.05]	0.001 *	− 0.66	− 0.10 [− 0.21, 0.01]	0.067	− 0.50
Inferior occipital gyrus	− 0.14 [− 0.23, − 0.05]	0.002 *	− 0.68	− 0.14 [− 0.25, − 0.02]	0.022	− 0.63
Cuneus	− 0.12 [− 0.20, − 0.03]	0.006 *	− 0.56	− 0.12 [− 0.23, − 0.02]	0.023	− 0.62
Calcarine fissure and surrounding cortex	− 0.16 [− 0.27, − 0.06]	0.002 *	− 0.65	− 0.19 [− 0.32, − 0.07]	0.004 *	− 0.83
Lingual gyrus	− 0.11 [− 0.19, − 0.03]	0.010 *	− 0.57	− 0.12 [− 0.23, − 0.01]	0.027	− 0.63
Fusiform gyrus	− 0.10 [− 0.18, − 0.01]	0.031 *	− 0.48	− 0.14 [− 0.25, − 0.02]	0.023	− 0.66
Insula	− 0.09 [− 0.16, − 0.02]	0.016 *	− 0.51	− 0.04 [− 0.14, 0.06]	0.445	− 0.23
*Right hemisphere*						
Rolandic operculum	− 0.08 [− 0.14, − 0.02]	0.008 *	− 0.57	− 0.07 [− 0.16, 0.01]	0.091	− 0.48
Superior frontal gyrus, orbital part	− 0.11 [− 0.21, − 0.01]	0.034 *	− 0.47	− 0.17 [− 0.31, − 0.02]	0.023	− 0.68
Inferior frontal gyrus, orbital part	− 0.08 [− 0.16, − 0.01]	0.037 *	− 0.47	− 0.09 [− 0.20, 0.03]	0.146	− 0.46
Olfactory cortex	− 0.10 [− 0.19, 0.00]	0.046 *	− 0.43	− 0.10 [− 0.24, 0.05]	0.175	− 0.39
Inferior temporal gyrus	− 0.17 [− 0.30, − 0.03]	0.018 *	− 0.53	− 0.14 [− 0.33, 0.04]	0.133	− 0.47
Inferior occipital gyrus	− 0.12 [− 0.23, − 0.02]	0.018 *	− 0.51	− 0.12 [− 0.27, 0.04]	0.132	− 0.48

Estimated differences (mm), 95% confidence intervals (CI), *p*-values, and effect sizes (*β*) for the exposure group component of models of cortical thickness. Model 1 accounts for sex, age, income, and caregiver education. Model 2 (male cohort) accounts for age, income, and total brain volume. * significant at 15% FDR

### Subcortical structures

An age by exposure interaction was found in the volume of the bilateral amygdalae (estimated difference = − 0.06 cm^3^/year, 95% CI [− 0.11, − 0.01], *p* = 0.011; *β* = − 0.39) (Fig. 4). Volumes in CHUU increased with age (estimated difference = 0.05 cm^3^, 95% CI [0.01, 0.09], *p* = 0.020, *β* = 0.31) but remained flat in CHEU (estimated difference = − 0.01 cm^3^, 95% CI [− 0.04, 0.02], *p* = 0.387, *β* = − 0.08). Male CHEU had smaller bilateral hippocampal volumes than male CHUU (estimated difference = − 0.21 cm^3^, 95% CI [− 0.40, − 0.01], *p* = 0.036; *β* = − 0.52) (Fig. 4); no difference was found in the female cohort (estimated difference = 0.01 cm^3^, 95% CI [− 0.21, 0.23], *p* = 0.925; *β* = 0.03).

## Discussion

The present study is the first to examine the neuroanatomic developmental consequences of in utero HIV and ART exposure among 6- to 12-year-old children. CHEU exhibited widespread decreases in brain volume and cortical thickness, with some regions more affected than others. Total brain volumes were smaller in CHEU within the full cohort and female cohort, but not the male cohort. Specific regional effects (incorporating total brain volume as a covariate) were found most frequently in the male cohort.

In three adjacent areas of the frontal lobe, the bilateral pars opercularis, left rolandic operculum, and left precentral gyrus, we found age by exposure interactions with similar patterns of convergence in volume between exposure groups over time. The pars opercularis is associated with phonological and semantic output, auditory self-monitoring, and speech production more broadly (in the left hemisphere), and cognitive inhibition and task-switching (in the right hemisphere) [38–42]. The rolandic operculum is associated with expressive language and speech production [43, 44], and sensory-auditory integration [43]. The precentral gyrus encompasses the primary motor and supplementary motor areas, and is key in the planning and control of voluntary movements [45, 46]. Areas near the midpoint of the precentral gyrus are implicated in speech motor planning and verbal fluency [47]. In typically developing children, the pars opercularis, rolandic operculum, and precentral gyrus show a growth trajectory that peaks in early childhood [48]; our findings suggest that these structures may be immature in CHEU compared to CHUU, which could result in deficits or delays in motor function, expressive language, and potentially receptive language.

The temporal lobes, smaller in male CHEU in our study, have wide-ranging functional associations, including language production and comprehension [49–52] and declarative memory [53–56]. Through the hippocampi, parahippocampal gyri, and temporal poles, they participate in a limbic circuit controlling memory and emotion, alongside the amygdalae and orbitofrontal cortex [57–60], both of which also showed exposure effects. The orbitofrontal cortex was found to be thinner in CHEU and is involved in reward value-based prediction, decision-making, and action selection [61–67]. The amygdalae, where we observed an age by exposure interaction, are well-known to be implicated in emotion processing, especially fear and anxiety [68, 69]. Growth of the amygdalae over this age range has been reported in typically developing children [70, 71], consistent with our observation of increased amygdalar volume with age in CHUU. The absence of this trend in CHEU suggests reduced growth or an altered growth trajectory of this structure. The hippocampi, observed to be smaller in male CHEU, play an important role in episodic memory consolidation [72–74]. Both hippocampi and amygdalae are involved in the mediation of anxiety associated with reward and punishment and the formation of reward-based memory [58, 75, 76]. Taken together, the above pattern of regional increases and decreases could result in alterations in memory, emotion, and decision-making.

The pattern of morphological differences between exposure groups in our study may explain some of the deficits or delays in motor function, speech and language, memory, decision-making, and emotion regulation observed in CHEU. Between 1 and 2 years, CHEU show delays in language and motor development [4, 6–8]. Some studies in the 1–4-year age range find cognitive and motor delays [5, 10], while others show increasing language impairments from 3 to 5 years [9, 10]. By 5 to 12 years, a range of deficits are detected in cognitive, language, math, memory, attention, and visuomotor function [11, 12, 14]. A meta-analysis of cognitive and motor deficits among 0–8-year-old CHEU suggests an association with ART exposure [13], and animal models isolating ART from HIV exposure show that both could contribute to these impairments [15, 16].

Although some CHEU have shown altered white matter integrity in infancy and early childhood [17, 18], other research has found no differences in white matter volume [15]. Our study conforms to the latter, finding no white matter volume differences. At 2–6 weeks of age, CHEU have exhibited reduced gray matter and caudate volumes [19]. A gray matter difference was not observed in the present study, perhaps due to the substantial changes in gray matter volume that occur in early childhood [48]. Caudate volumes did not differ between exposure groups in our study, but other subcortical regions (amygdalae and hippocampi) were affected.

Increased prefrontal cortical thickness—especially in the medial orbitofrontal cortex—has been reported in 2–3-year-old CHEU [20]. Our study reproduced the location of regional changes, but not the direction of the effect: overall cortical thickness and prefrontal and orbitofrontal cortical thickness were decreased in CHEU. Left hemisphere occipital differences were also more prominent in our analysis. Differences in study setting, maternal treatment, viral load, CD4 count, and particularly child age at assessment likely contributed to these differences, emphasizing the need for continued follow-up of these high-risk children.

While the mechanisms leading to altered brain development in CHEU are presently unknown, the pattern of affected regions offers insight. The most striking observation was a largely uniform effect on brain growth, with few regions showing deviations unexplained by overall brain volume changes. This suggests that these changes occur at an early stage of in utero development, starting in the first trimester before much regional specialization has occurred.

The specific regions that were differentially affected also offer insights. We detected an age by exposure interaction in the volume of the amygdalae, characterized by a significant increase over time only in CHUU, suggesting an effect of in utero HIV or ART exposure. The amygdalae’s most significant period of development is between 12 and 16 weeks of gestation, with all major nuclei fully formed at ~ 15 weeks [77, 78]. The amygdalae are especially sensitive to stress and adversity, with high stress associated with higher amygdalar volumes [79–81] in early [77, 82] and late [83] pregnancy. It has been reported that people living with HIV experience high rates of anxiety, depression, and stress [84, 85], more than their HIV-negative counterparts [86, 87], and pregnant people living with HIV are especially prone to these conditions, with social stigma and socioeconomic status as common factors [88–91]. Prenatal depression is associated with alterations in limbic network connectivity and the amygdalar, hippocampal, and frontal cortical structure of offspring, extending into adolescence [92]. Prenatal distress has also been linked to infant microstructural and functional connectivity between the amygdalae and prefrontal cortex [93]. A potential mechanism for this effect is the movement of cortisol across the placenta, which can then bind to glucocorticoid receptors in the brain. This signaling can influence neurodevelopment via the hypothalamic–pituitary–adrenal axis, responsible for stress hormone production and linked to both amygdalar and hippocampal development. There is some evidence to show these effects are mediated by sex hormones [94, 95]. Stress that the fetus may have experienced by developing in an environment impacted by HIV infection could also have similar impacts on the amygdalae. It is notable that of all regions of significance in the current study, the amygdalae exhibited a pattern that could be consistent with earlier maturation. Studies of subcortical volumes associated with stress and adversity find increases in amygdalar volume [96] but decreases in areas such as the hippocampi [97]. Our hippocampal findings also showed a volume decrease among CHEU. The hippocampi are distinguishable from other regions by 8 and 9 weeks of gestation, and peak neurogenesis occurs between 16 and 20 weeks [97]. The early stage of pregnancy is critical for both hippocampal and amygdalar development, and disruptions over this period may underlie both findings.

Prenatal adversity appears to produce more severe neurodevelopmental outcomes in males [98, 99], which comports with the number of sex-linked effects found in the current study. Male fetuses may be more vulnerable to adverse cognitive outcomes, including impairments in cognitive function, learning, and memory, and increased probability of neurodevelopmental disorders and learning disabilities [98–100]. There is some evidence that these differences stem from sex differences in placental adaptation to in utero stressors [100].

In utero exposure to HIV and ART can disrupt neurodevelopment through multiple interrelated mechanisms. Different ART regimens have shown different long-term neurodevelopmental outcomes at up to 12 years [101], and the timing of ART initiation during pregnancy has been connected to alterations in white matter microstructure of limbic and subcortical brain regions in CHEU [102]. In utero HIV/ART exposure is associated with placental disruptions such as maternal vascular malperfusion (MVM) and both acute and chronic inflammation, all of which may contribute to preterm birth and restricted fetal growth [103]. MVM is associated with ART exposure from conception, in contrast with initiation during pregnancy [104]. One possible mechanism may be the interaction between inflammatory cytokines and angiogenic factors critical to placental development [103]. Despite viral suppression with ART, PLWH show persistently elevated levels of pro-inflammatory cytokines. ART may not suppress all immune dysregulation, potentially allowing HIV to affect transplacental antibody transfer and compounding risks for adverse neurodevelopmental outcomes [105]. Immune system dysregulation and chronic systemic inflammation, characterized by persistently elevated pro-inflammatory cytokines, are also heightened in CHEU [106–108]. These cytokines can cross the blood–brain barrier, disrupting neurodevelopment and increasing the risk of cognitive deficits [109].

Despite the novelty of our study, there are some limitations. While the relatively low viral load during pregnancy reported among birth parents may have reduced the in utero effect of HIV exposure, potentially emphasizing the ART exposure effect, the heterogeneity of ART exposure and relatively small sample size limited our ability to investigate specific ART class effects. Future analyses (upon further recruitment) will assess these effects. Although the difference was not significant, the household income of CHUU was higher than that of CHEU. We corrected for this difference by including income as a covariate in all models. We acknowledge the imbalance in sex distribution between the CHEU group (53% female) and the CHUU group (39% female) and its potential impact on the interpretation of sex differences. Although sex-specific effects were observed, the limited sample size in the stratified analyses restricts the generalizability of the findings. These results should therefore be interpreted with caution; we hope to verify these findings in the future with a larger and more balanced cohort. Differences between CHEU and CHUU in birth weight, gestational age at birth, and prematurity may partially explain observed neurodevelopmental outcomes, but these variables likely functioned as mediators of the in utero HIV/ART exposure effect on neurodevelopment, rather than confounders. They were excluded from the models’ covariates to avoid distorting said effect. Unfortunately, our sample size precluded mediation analysis. We acknowledge the potential for recruitment bias of CHEU and CHUU, as a parent’s concerns for their child’s neurodevelopment may increase their likelihood to participate in the study. The cross-sectional nature of the study cannot exclude factors that change with time and would appear colinear with age in our analysis.

## Conclusions

HIV/ART in utero exposure is associated with significantly reduced brain volume and cortical thickness in CHEU, thus showing considerable neurodevelopmental impacts. These MRI findings extend prior reports of morphological changes at younger ages and reduced performance on neuropsychological testing batteries. These findings reinforce the need for additional research in this under-resourced and growing population, and the need to explore early interventions to support healthy brain development in these children.

## Supplementary Information

Additional file 1: Appendix S1. STROBE Statement—Checklist of items that should be included in reports of cross-sectional studies.

Additional file 2: Supplementary Table S1. Regional neuroanatomical measurements (cortical volume, thickness, surface area, and subcortical volume) for each participant.

Additional file 3: Supplementary Tables S2–S3: Estimated differences in regional cortical measurements by exposure group. Supplementary Table S2. Estimated differences in cortical volume by exposure group. Supplementary Table S3. Estimated differences in cortical thickness by exposure group.

## Data Availability

The datasets generated and analyzed during the current study are available from the corresponding author upon reasonable request. Supplementary Table S1 contains aggregated neuroanatomical measurements for each participant, including regional cortical volume, thickness, surface area, and subcortical volume.

## Abbreviations

AAL: Automated anatomical labeling
ART: Antiretroviral therapy
CHEU: Children who are HIV-exposed and uninfected
CHUU: Children who are HIV-unexposed and uninfected
FA: Flip angle
FDR: False discovery rate
FOV: Field of view
HIV: Human immunodeficiency virus
INSTI: Integrase strand transfer inhibitor
MAGeT: Multiple automatically generated templates
MFG: Middle frontal gyrus
MR(I): Magnetic resonance (imaging)
MVM: Maternal vascular malperfusion
NNRTI: Non-nucleoside reverse transcriptase inhibitor
NRTI: Nucleoside reverse transcriptase inhibitor
PI: Protease inhibitor
STROBE: Strengthening the Reporting of Observational Studies in Epidemiology
TE: Echo time
TR: Repetition time
