# Supplementary material for: Structural brain differences in school-aged children who are HIV-exposed uninfected
Source: BMC Med. 2025 Aug 26;23:496. doi: 10.1186/s12916-025-04332-3 (PMC12382074; doi:10.1186/s12916-025-04332-3)
Supplement: Supplementary file 2 — Additional file 2: Supplementary Table S1. Regional neuroanatomical measurements (cortical volume, thickness, surface area, and subcortical volume) for each participant. [file 12916_2025_4332_MOESM2_ESM.pdf]

Supplementary Table S2. Estimated differences in cortical volume by exposure group.

Region	Model 1 (entire cohort)			Model 2 (male cohort)		
	Estimated difference (mm) [95% CI]	*p*-value	β	Estimated difference (mm) [95% CI]	*p*-value	β
Left hemisphere						
Gyrus rectus	−0.16 [−0.32, −0.01]	0.042 *	−0.45	0.04 [−0.12, 0.21]	0.600	0.12
Olfactory cortex	−0.04 [−0.07, −0.01]	0.007 *	−0.58	−0.01 [−0.04, 0.03]	0.598	−0.13
Superior frontal gyrus, orbital part	−0.20 [−0.53, 0.12]	0.220	−0.27	0.06 [−0.34, 0.45]	0.775	0.07
Superior frontal gyrus, medial orbital	−0.18 [−0.40, 0.03]	0.088	−0.38	0.07 [−0.17, 0.31]	0.567	0.14
Middle frontal gyrus, orbital part	−0.09 [−0.27, 0.10]	0.369	−0.20	−0.04 [−0.30, 0.21]	0.733	−0.10
Inferior frontal gyrus, orbital part	−0.32 [−0.58, −0.07]	0.015 *	−0.53	−0.17 [−0.48, 0.15]	0.286	−0.29
Superior frontal gyrus, dorsolateral	−0.42 [−1.14, 0.29]	0.243	−0.23	0.33 [−0.54, 1.19]	0.446	0.19
Middle frontal gyrus	−1.18 [−2.16, −0.21]	0.018 *	−0.51	0.32 [−0.59, 1.24]	0.481	0.15
Inferior frontal gyrus, opercular part	−0.27 [−0.47, −0.06]	0.010 *	−0.54	−0.15 [−0.41, 0.11]	0.244	−0.30
Inferior frontal gyrus, triangular part	−0.61 [−1.00, −0.22]	0.003 *	−0.64	−0.31 [−0.80, 0.18]	0.214	−0.32
Superior frontal gyrus, medial	−0.32 [−0.88, 0.24]	0.262	−0.24	0.10 [−0.55, 0.76]	0.753	0.08
Supplementary motor area	0.10 [−0.40, 0.60]	0.695	0.09	0.31 [−0.41, 1.02]	0.388	0.25
Paracentral lobule	0.01 [−0.40, 0.42]	0.970	0.01	0.23 [−0.34, 0.80]	0.416	0.24
Precentral gyrus	−0.38 [−1.13, 0.37]	0.317	−0.22	0.15 [−0.75, 1.06]	0.734	0.09
Rolandic operculum	−0.27 [−0.49, −0.04]	0.021 *	−0.48	−0.17 [−0.45, 0.11]	0.218	−0.32
Postcentral gyrus	−0.21 [−1.00, 0.57]	0.588	−0.11	0.35 [−0.52, 1.21]	0.419	0.20
Superior parietal gyrus	−0.47 [−1.30, 0.36]	0.265	−0.25	−0.47 [−1.49, 0.54]	0.351	−0.26
Supramarginal gyrus	−0.29 [−1.04, 0.46]	0.441	−0.16	0.01 [−0.81, 0.82]	0.985	0.01
Angular gyrus	−0.70 [−1.34, −0.06]	0.032 *	−0.44	−0.55 [−1.39, 0.29]	0.191	−0.34
Precuneus	−0.49 [−0.97, −0.004]	0.048 *	−0.37	−0.18 [−0.71, 0.35]	0.496	−0.16
Superior occipital gyrus	−0.34 [−0.68, 0.004]	0.053 *	−0.39	−0.19 [−0.66, 0.28]	0.422	−0.23
Middle occipital gyrus	−0.71 [−1.38, −0.04]	0.038 *	−0.43	−0.43 [−1.31, 0.46]	0.336	−0.28
Inferior occipital gyrus	−0.27 [−0.51, −0.02]	0.031 *	−0.43	−0.26 [−0.59, 0.07]	0.121	−0.44
Calcarine fissure and surrounding cortex	−0.37 [−0.94, 0.20]	0.196	−0.28	−0.28 [−1.03, 0.47]	0.460	−0.21
Cuneus	−0.29 [−0.71, 0.13]	0.175	−0.27	−0.02 [−0.61, 0.58]	0.957	−0.02
Lingual gyrus	−0.38 [−0.70, −0.07]	0.018 *	−0.48	−0.25 [−0.68, 0.17]	0.234	−0.33
Fusiform gyrus	−0.62 [−1.10, −0.14]	0.011 *	−0.48	−0.50 [−1.01, 0.01]	0.054	−0.45
Heschl’s gyrus	−0.11 [−0.23, 0.02]	0.095	−0.36	0.01 [−0.14, 0.16]	0.900	0.03
Superior temporal gyrus	−1.30 [−2.12, −0.49]	0.002 *	−0.61	−0.90 [−1.65, −0.15]	0.020	−0.44
Middle temporal gyrus	−0.96 [−1.74, −0.18]	0.016 *	−0.46	−0.87 [−1.61, −0.12]	0.024	−0.48
Inferior temporal gyrus	−0.32 [−0.99, 0.34]	0.336	−0.20	−0.33 [−1.06, 0.41]	0.371	−0.22
Temporal pole, superior temporal gyrus	−0.36 [−0.62, −0.09]	0.009 *	−0.51	−0.06 [−0.39, 0.27]	0.711	−0.10
Temporal pole, middle temporal gyrus	−0.20 [−0.34, −0.06]	0.005 *	−0.56	−0.11 [−0.27, 0.05]	0.180	−0.36
Parahippocampal gyrus	−0.27 [−0.48, −0.07]	0.008 *	−0.53	−0.18 [−0.42, 0.06]	0.146	−0.40
Anterior cingulate and paracingulate gyri	−0.08 [−0.28, 0.13]	0.457	−0.17	0.12 [−0.07, 0.31]	0.194	0.29
Median cingulate and paracingulate gyri	−0.10 [−0.34, 0.14]	0.402	−0.18	−0.02 [−0.30, 0.26]	0.909	−0.03

Posterior cingulate gyrus	−0.09 [−0.18, 0.003]	0.058 *	−0.37	−0.07 [−0.19, 0.05]	0.227	−0.33
Insula	−0.28 [−0.52, −0.03]	0.028 *	−0.45	−0.09 [−0.36, 0.18]	0.496	−0.16
Right hemisphere						
Gyrus rectus	−0.14 [−0.30, 0.02]	0.090	−0.37	−0.02 [−0.19, 0.15]	0.835	−0.05
Olfactory cortex	−0.03 [−0.06, 0.002]	0.067 *	−0.40	−0.003 [−0.04, 0.03]	0.856	−0.04
Superior frontal gyrus, orbital part	−0.31 [−0.64, 0.02]	0.067 *	−0.40	−0.10 [−0.44, 0.24]	0.561	−0.13
Superior frontal gyrus, medial orbital	−0.17 [−0.39, 0.04]	0.113	−0.34	0.0004 [−0.27, 0.27]	0.998	0.00
Middle frontal gyrus, orbital part	−0.12 [−0.31, 0.08]	0.234	−0.27	−0.04 [−0.29, 0.21]	0.745	−0.08
Inferior frontal gyrus, orbital part	−0.17 [−0.47, 0.13]	0.259	−0.25	0.009 [−0.33, 0.35]	0.955	0.01
Superior frontal gyrus, dorsolateral	−0.34 [−1.03, 0.35]	0.329	−0.21	0.39 [−0.39, 1.18]	0.318	0.26
Middle frontal gyrus	−0.64 [−1.76, 0.48]	0.261	−0.24	0.49 [−0.62, 1.60]	0.376	0.20
Inferior frontal gyrus, opercular part	−0.18 [−0.37, 0.01]	0.063 *	−0.40	−0.04 [−0.23, 0.15]	0.665	−0.11
Inferior frontal gyrus, triangular part	−0.16 [−0.56, 0.24]	0.435	−0.17	0.17 [−0.20, 0.54]	0.358	0.24
Superior frontal gyrus, medial	−0.49 [−1.14, 0.16]	0.141	−0.33	0.15 [−0.51, 0.81]	0.652	0.11
Supplementary motor area	0.06 [−0.32, 0.45]	0.749	0.07	0.34 [−0.11, 0.79]	0.134	0.43
Paracentral lobule	0.05 [−0.32, 0.41]	0.796	0.05	0.27 [−0.21, 0.74]	0.269	0.32
Precentral gyrus	−0.26 [−0.95, 0.44]	0.464	−0.15	0.11 [−0.80, 1.02]	0.810	0.06
Rolandic operculum	−0.22 [−0.44, −0.01]	0.043 *	−0.44	−0.16 [−0.39, 0.07]	0.171	−0.33
Postcentral gyrus	−0.22 [−0.93, 0.49]	0.539	−0.13	0.14 [−0.61, 0.89]	0.702	0.08
Superior parietal gyrus	−0.74 [−1.46, −0.03]	0.043 *	−0.45	−0.40 [−1.22, 0.41]	0.320	−0.27
Supramarginal gyrus	−0.37 [−1.05, 0.30]	0.276	−0.23	−0.46 [−1.14, 0.22]	0.176	−0.30
Angular gyrus	−0.79 [−1.36, −0.21]	0.008 *	−0.54	−0.35 [−1.08, 0.38]	0.340	−0.25
Precuneus	−0.56 [−1.07, −0.04]	0.036 *	−0.41	−0.14 [−0.69, 0.40]	0.601	−0.13
Superior occipital gyrus	−0.53 [−0.92, −0.15]	0.007 *	−0.55	−0.26 [−0.81, 0.29]	0.340	−0.30
Middle occipital gyrus	−1.30 [−2.10, −0.49]	0.002 *	−0.62	−0.65 [−1.79, 0.50]	0.258	−0.33
Inferior occipital gyrus	−0.44 [−0.77, −0.10]	0.011 *	−0.50	−0.33 [−0.78, 0.13]	0.152	−0.37
Calcarine fissure and surrounding cortex	−0.35 [−0.95, 0.26]	0.262	−0.24	−0.39 [−1.14, 0.36]	0.299	−0.27
Cuneus	−0.52 [−0.97, −0.08]	0.022 *	−0.46	−0.36 [−0.93, 0.20]	0.203	−0.36
Lingual gyrus	−0.29 [−0.60, 0.03]	0.073 *	−0.36	−0.29 [−0.70, 0.12]	0.158	−0.38
Fusiform gyrus	−0.66 [−1.14, −0.17]	0.009 *	−0.50	−0.53 [−1.18, 0.12]	0.110	−0.44
Heschl's gyrus	−0.09 [−0.20, 0.02]	0.124	−0.33	−0.08 [−0.21, 0.06]	0.256	−0.30
Superior temporal gyrus	−0.68 [−1.42, 0.05]	0.067 *	−0.38	−0.39 [−1.16, 0.39]	0.323	−0.24
Middle temporal gyrus	−1.27 [−2.10, −0.45]	0.003 *	−0.57	−0.69 [−1.48, 0.11]	0.088	−0.36
Inferior temporal gyrus	−0.76 [−1.43, −0.09]	0.028 *	−0.44	−0.46 [−1.23, 0.31]	0.236	−0.29
Temporal pole, superior temporal gyrus	−0.11 [−0.41, 0.20]	0.488	−0.15	0.07 [−0.27, 0.40]	0.688	0.11
Temporal pole, middle temporal gyrus	−0.08 [−0.24, 0.09]	0.356	−0.20	−0.05 [−0.25, 0.14]	0.574	−0.16
Parahippocampal gyrus	−0.26 [−0.44, −0.07]	0.008 *	−0.52	−0.29 [−0.51, −0.07]	0.011	−0.71
Anterior cingulate and paracingulate gyri	−0.16 [−0.34, 0.02]	0.081	−0.39	0.01 [−0.15, 0.18]	0.865	0.03
Median cingulate and paracingulate gyri	−0.16 [−0.40, 0.09]	0.205	−0.28	0.01 [−0.20, 0.22]	0.925	0.02
Posterior cingulate gyrus	−0.10 [−0.20, 0.01]	0.069 *	−0.37	−0.05 [−0.17, 0.08]	0.432	−0.20
Insula	−0.12 [−0.38, 0.14]	0.381	−0.18	0.08 [−0.17, 0.33]	0.500	0.15

2

Estimated differences (mm), 95% confidence intervals (CI), *p*-values, and effect sizes (β) for the exposure group component of models of cortical volume. Model 1 accounts for sex, age, income, and caregiver education. Model 2 (male cohort) accounts for age, income, and total brain volume. * significant at 15% FDR.

Supplementary Table S3. Estimated differences in cortical thickness by exposure group.

Region	Model 1 (entire cohort)			Model 2 (male cohort)		
	Estimated difference (mm) [95% CI]	*p*-value	β	Estimated difference (mm) [95% CI]	*p*-value	β
Left hemisphere						
Gyrus rectus	−0.16 [−0.30, −0.02]	0.023 *	−0.49	−0.13 [−0.34, 0.08]	0.231	−0.36
Olfactory cortex	−0.20 [−0.33, −0.07]	0.003 *	−0.63	−0.14 [−0.33, 0.06]	0.170	−0.41
Superior frontal gyrus, orbital part	−0.15 [−0.26, −0.03]	0.011 *	−0.55	−0.16 [−0.32, 0.01]	0.060	−0.58
Superior frontal gyrus, medial orbital	−0.13 [−0.22, −0.03]	0.009 *	−0.54	−0.11 [−0.25, 0.02]	0.095	−0.46
Middle frontal gyrus, orbital part	−0.10 [−0.20, −0.003]	0.044 *	−0.43	−0.20 [−0.32, −0.07]	0.002	−0.86
Inferior frontal gyrus, orbital part	−0.09 [−0.15, −0.02]	0.010 *	−0.51	−0.11 [−0.20, −0.01]	0.031	−0.60
Superior frontal gyrus, dorsolateral	−0.09 [−0.18, −0.01]	0.038 *	−0.44	−0.09 [−0.22, 0.03]	0.125	−0.44
Middle frontal gyrus	−0.07 [−0.14, −0.004]	0.039 *	−0.42	−0.04 [−0.14, 0.06]	0.400	−0.23
Inferior frontal gyrus, opercular part	−0.03 [−0.10, 0.03]	0.322	−0.22	−0.03 [−0.13, 0.07]	0.570	−0.17
Inferior frontal gyrus, triangular part	−0.04 [−0.10, 0.02]	0.180	−0.27	−0.03 [−0.11, 0.06]	0.549	−0.17
Superior frontal gyrus, medial	−0.07 [−0.16, 0.01]	0.097	−0.34	−0.06 [−0.18, 0.06]	0.308	−0.28
Supplementary motor area	−0.01 [−0.10, 0.07]	0.755	−0.07	−0.02 [−0.13, 0.09]	0.665	−0.13
Paracentral lobule	−0.06 [−0.14, 0.02]	0.155	−0.31	−0.08 [−0.18, 0.02]	0.112	−0.48
Precentral gyrus	−0.04 [−0.11, 0.02]	0.191	−0.29	−0.06 [−0.15, 0.03]	0.166	−0.41
Rolandic operculum	−0.07 [−0.14, −0.01]	0.022 *	−0.49	−0.09 [−0.18, −0.004]	0.040	−0.57
Postcentral gyrus	−0.06 [−0.14, 0.01]	0.112	−0.35	−0.04 [−0.15, 0.07]	0.456	−0.23
Superior parietal gyrus	−0.04 [−0.10, 0.02]	0.195	−0.28	−0.05 [−0.13, 0.03]	0.245	−0.34
Supramarginal gyrus	−0.03 [−0.09, 0.04]	0.419	−0.17	−0.05 [−0.14, 0.03]	0.216	−0.34
Angular gyrus	−0.08 [−0.14, −0.02]	0.007 *	−0.55	−0.12 [−0.21, −0.04]	0.006	−0.78
Precuneus	−0.05 [−0.11, 0.02]	0.176	−0.28	−0.04 [−0.12, 0.04]	0.307	−0.31
Superior occipital gyrus	−0.12 [−0.22, −0.03]	0.013 *	−0.48	−0.09 [−0.23, 0.06]	0.235	−0.35
Middle occipital gyrus	−0.13 [−0.21, −0.05]	0.001 *	−0.66	−0.10 [−0.21, 0.01]	0.067	−0.50
Inferior occipital gyrus	−0.14 [−0.23, −0.05]	0.002 *	−0.68	−0.14 [−0.25, −0.02]	0.022	−0.63
Calcarine fissure and surrounding cortex	−0.16 [−0.27, −0.06]	0.002 *	−0.65	−0.19 [−0.32, −0.07]	0.004	−0.83
Cuneus	−0.12 [−0.20, −0.03]	0.006 *	−0.56	−0.12 [−0.23, −0.02]	0.023	−0.62
Lingual gyrus	−0.11 [−0.19, −0.03]	0.010 *	−0.57	−0.12 [−0.23, −0.01]	0.027	−0.63

Fusiform gyrus	−0.10 [−0.18, −0.01]	0.031 *	−0.48	−0.14 [−0.25, −0.02]	0.023	−0.66
Heschl's gyrus	−0.08 [−0.15, −0.01]	0.032 *	−0.46	−0.05 [−0.16, 0.05]	0.322	−0.29
Superior temporal gyrus	−0.08 [−0.16, −0.004]	0.041 *	−0.44	−0.09 [−0.21, 0.03]	0.150	−0.42
Middle temporal gyrus	−0.08 [−0.17, 0.01]	0.070	−0.40	−0.10 [−0.23, 0.03]	0.123	−0.47
Inferior temporal gyrus	−0.09 [−0.22, 0.03]	0.126	−0.34	−0.10 [−0.27, 0.07]	0.240	−0.37
Temporal pole, superior temporal gyrus	−0.12 [−0.24, 0.01]	0.070	−0.40	−0.02 [−0.21, 0.16]	0.792	−0.08
Temporal pole, middle temporal gyrus	−0.13 [−0.30, 0.03]	0.105	−0.36	−0.04 [−0.28, 0.21]	0.764	−0.09
Parahippocampal gyrus	−0.06 [−0.14, 0.03]	0.204	−0.28	−0.08 [−0.20, 0.03]	0.138	−0.43
Anterior cingulate and paracingulate gyri	−0.04 [−0.12, 0.05]	0.387	−0.18	0.01 [−0.12, 0.13]	0.920	0.03
Median cingulate and paracingulate gyri	−0.03 [−0.11, 0.05]	0.470	−0.15	0.003 [−0.10, 0.11]	0.957	0.02
Posterior cingulate gyrus	−0.03 [−0.14, 0.07]	0.506	−0.14	−0.02 [−0.16, 0.11]	0.720	−0.11
Insula	−0.09 [−0.16, −0.02]	0.016 *	−0.51	−0.04 [−0.14, 0.06]	0.445	−0.23
Right hemisphere						
Gyrus rectus	−0.07 [−0.19, 0.05]	0.242	−0.26	−0.07 [−0.25, 0.10]	0.407	−0.25
Olfactory cortex	−0.10 [−0.19, −0.002]	0.046 *	−0.43	−0.10 [−0.24, 0.05]	0.175	−0.39
Superior frontal gyrus, orbital part	−0.11 [−0.21, −0.01]	0.034 *	−0.47	−0.17 [−0.31, −0.02]	0.023	−0.68
Superior frontal gyrus, medial orbital	−0.09 [−0.18, 0.002]	0.054	−0.41	−0.10 [−0.22, 0.02]	0.102	−0.47
Middle frontal gyrus, orbital part	−0.09 [−0.19, 0.02]	0.107	−0.36	−0.14 [−0.28, 0.001]	0.052	−0.59
Inferior frontal gyrus, orbital part	−0.08 [−0.16, −0.01]	0.037 *	−0.47	−0.09 [−0.20, 0.03]	0.146	−0.46
Superior frontal gyrus, dorsolateral	−0.06 [−0.15, 0.03]	0.172	−0.29	−0.08 [−0.20, 0.05]	0.214	−0.37
Middle frontal gyrus	−0.04 [−0.11, 0.03]	0.300	−0.22	−0.03 [−0.13, 0.07]	0.530	−0.18
Inferior frontal gyrus, opercular part	−0.06 [−0.12, 0.01]	0.100	−0.37	−0.04 [−0.13, 0.05]	0.393	−0.25
Inferior frontal gyrus, triangular part	−0.05 [−0.12, 0.01]	0.099	−0.35	−0.06 [−0.14, 0.03]	0.183	−0.39
Superior frontal gyrus, medial	−0.08 [−0.17, 0.01]	0.096	−0.35	−0.06 [−0.19, 0.08]	0.408	−0.23
Supplementary motor area	−0.02 [−0.10, 0.06]	0.624	−0.10	−0.03 [−0.14, 0.08]	0.549	−0.18
Paracentral lobule	−0.05 [−0.12, 0.02]	0.145	−0.31	−0.05 [−0.14, 0.05]	0.334	−0.28
Precentral gyrus	−0.06 [−0.12, 0.003]	0.065	−0.40	−0.05 [−0.13, 0.04]	0.281	−0.33
Rolandic operculum	−0.08 [−0.14, −0.02]	0.008 *	−0.57	−0.07 [−0.16, 0.01]	0.091	−0.48
Postcentral gyrus	−0.03 [−0.10, 0.05]	0.521	−0.14	−0.02 [−0.12, 0.09]	0.729	−0.10
Superior parietal gyrus	−0.04 [−0.12, 0.03]	0.277	−0.24	−0.04 [−0.13, 0.05]	0.411	−0.26
Supramarginal gyrus	−0.02 [−0.09, 0.05]	0.632	−0.11	−0.03 [−0.11, 0.06]	0.551	−0.17
Angular gyrus	−0.03 [−0.11, 0.04]	0.376	−0.19	−0.02 [−0.13, 0.10]	0.793	−0.08
Precuneus	−0.05 [−0.11, 0.02]	0.158	−0.31	−0.01 [−0.09, 0.07]	0.792	−0.08
Superior occipital gyrus	−0.06 [−0.15, 0.03]	0.180	−0.27	0.05 [−0.07, 0.17]	0.378	0.26
Middle occipital gyrus	−0.07 [−0.15, 0.01]	0.090	−0.36	−0.02 [−0.13, 0.09]	0.702	−0.12
Inferior occipital gyrus	−0.12 [−0.23, −0.02]	0.018 *	−0.51	−0.12 [−0.27, 0.04]	0.132	−0.48
Calcarine fissure and surrounding cortex	−0.08 [−0.17, 0.01]	0.064	−0.38	−0.04 [−0.15, 0.06]	0.393	−0.24
Cuneus	−0.07 [−0.14, 0.002]	0.056	−0.39	−0.02 [−0.11, 0.06]	0.604	−0.15
Lingual gyrus	−0.06 [−0.14, 0.01]	0.100	−0.36	−0.06 [−0.15, 0.04]	0.221	−0.35
Fusiform gyrus	−0.09 [−0.19, 0.01]	0.080	−0.40	−0.12 [−0.24, −0.01]	0.039	−0.60

Heschl's gyrus	−0.06 [−0.14, 0.02]	0.133	−0.33	−0.04 [−0.13, 0.05]	0.362	−0.26
Superior temporal gyrus	−0.03 [−0.11, 0.06]	0.523	−0.14	−0.04 [−0.14, 0.06]	0.445	−0.22
Middle temporal gyrus	−0.08 [−0.18, 0.03]	0.150	−0.32	−0.08 [−0.23, 0.08]	0.316	−0.32
Inferior temporal gyrus	−0.17 [−0.30, −0.03]	0.018 *	−0.53	−0.14 [−0.33, 0.04]	0.133	−0.47
Temporal pole, superior temporal gyrus	−0.02 [−0.14, 0.11]	0.775	−0.07	−0.05 [−0.22, 0.12]	0.561	−0.18
Temporal pole, middle temporal gyrus	−0.11 [−0.30, 0.08]	0.263	−0.25	−0.17 [−0.43, 0.09]	0.191	−0.41
Parahippocampal gyrus	−0.06 [−0.16, 0.03]	0.181	−0.30	−0.12 [−0.24, −0.002]	0.046	−0.58
Anterior cingulate and paracingulate gyri	−0.04 [−0.12, 0.05]	0.405	−0.19	−0.03 [−0.16, 0.10]	0.630	−0.14
Median cingulate and paracingulate gyri	−0.05 [−0.12, 0.01]	0.104	−0.34	−0.03 [−0.11, 0.05]	0.482	−0.21
Posterior cingulate gyrus	−0.03 [−0.13, 0.06]	0.505	−0.14	0.02 [−0.10, 0.15]	0.696	0.12
Insula	−0.05 [−0.12, 0.01]	0.119	−0.35	−0.003 [−0.09, 0.08]	0.943	−0.02

Estimated differences (mm), 95% confidence intervals (CI), *p*-values, and effect sizes (β) for the exposure group component of models of cortical thickness. Model 1 accounts for sex, age, income, and caregiver education. Model 2 (male cohort) accounts for age, income, and total brain volume. * significant at 15% FDR.
